# Segmentation of skin lesion using Cohen–Daubechies–Feauveau biorthogonal wavelet

**DOI:** 10.1186/s40064-016-3211-4

**Published:** 2016-09-19

**Authors:** Shehzad Khalid, Uzma Jamil, Kashif Saleem, M. Usman Akram, Waleed Manzoor, Waqas Ahmed, Amina Sohail

**Affiliations:** 1 Department of Computer Engineering, Bahria University, Islamabad, Pakistan; 2 Government College University, Faisalabad, Pakistan; 3 National University of Sciences and Technology, Islamabad, Pakistan

**Keywords:** Image enhancement, Dermoscopy, Skin cancer, Border detection, Image segmentation

## Abstract

This paper presents a novel technique for segmentation of skin lesion in dermoscopic images based on wavelet transform along with morphological operations. The acquired dermoscopic images may include artifacts inform of gel, dense hairs and water bubble which make accurate segmentation more challenging. We have also embodied an efficient approach for artifacts removal and hair inpainting, to enhance the overall segmentation results. In proposed research, color space is also analyzed and selection of blue channel for lesion segmentation have confirmed better performance than techniques which utilizes gray scale conversion. We tackle the problem by finding the most suitable mother wavelet for skin lesion segmentation. The performance achieved with ‘bior6.8’ Cohen–Daubechies–Feauveau biorthogonal wavelet is found to be superior as compared to other wavelet family. The proposed methodology achieves 93.87 % accuracy on dermoscopic images of PH2 dataset acquired at Dermatology Service of Hospital Pedro Hispano, Matosinhos, Portugal.

## Background

Skin cancer is one of a major concern of world due to its alarming increasing rate (Day and Barbour 2000). The most common type of skin cancer is known as melanoma which originate in the pigment-producing melanocytes in bottom layer of skin. Melonoma mostly occurs on sun-exposed parts of the body because intermittent sun exposure damages the skin cells’ DNA with the ultraviolet (UV) radiation, as a result melanocytes become cancerous. However, it can also occur inside eye in iris and also in choroid layer. Melanoma can be classified into malignant melanoma and non-melanoma. Malignant melanoma is less common and accounts only for 5 % of skin cancers however it this is most aggressive, complex and fatal. According to American Cancer Society (ACS), approximately 10,130 people loss their life inside United States in year 2016 because of malignant melanoma (Siegel et al. 2016). The prevalence of melanoma raises every year. Melanoma detection of patients at early stage is of prime importance for the effective treatment. In later stages treatment become hard and melanoma can be fatal.

**Fig. 1:**
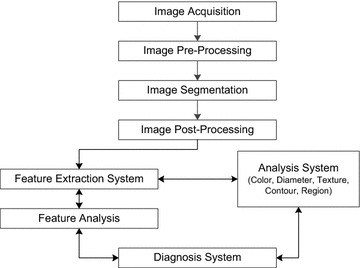
Computer added diagnostic steps

The procedure adopted for examining skin surface is known as dermoscopy or epiluminescence microscopy (ELM). It is a non-invasive method to screen out malignant melanoma by observing diagnostic features with special optical equipment, which is not possible with naked eye examination. Vestergaard et al. (2008) have reported mean sensitivity of 95 % with dermoscopy which is more accurate as compared to 74 % with naked eye examination in their comparative study for diagnosis of malignant melanoma. Nowadays, digital dermoscopy is preferred approach for examining the pigmented skin lesion by acquiring magnified and illuminated digital images. However, camera and other optical equipment specifications may influence the quality of acquired image as well as accuracy of diagnosis. The manual diagnosis of melanoma in its early stage depends on the availability of expert doctor with proper health care facilities and equipment. In many cases expert physicians are not available or they don’t have proper medical facilities to diagnose such kind of cancer at early stage. Therefore, automated computer added diagnostic (CAD) using digital dermoscopic images is extensively used from last decade for early diagnosis of skin cancer related disease. Automated CAD based system employ image processing along with machine learning techniques for the classification of skin lesion. These CAD systems performs automatic melanoma classification by using image processing based techniques as shown in Fig. 1. Recently, CAD system like health social networks (HSN) based on personal health profiles facilitates doctors and patients about medical decision making (Elmisery et al. 2015).

**Fig. 2:**
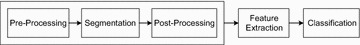
CAD process and segmentation break-up

Segmentation is one of the key step in CAD. In case of skin cancer detection, purpose of segmentation is to segment the effected part of the skin from the normal part. Better segmentation leads better features extraction, classification and diagnosis. Thus diagnostic system’s final results are highly dependent on the quality of segmentation performed. Segmentation could be fully automated (automated analysis end-to-end) or semi-automated (with some mouse clicks). Fully automated segmentation is a complex and challenging task to perform due to the versatility of dermoscopic images from multiple sources, variation of skin color and multiple artifacts like skin tones, hairs, gel, water bubbles or skin lines. The impact of these artifacts is minimized by using image pre-processing steps otherwise already mentioned artifacts can have negative impact on the feature computation and effects skin cancer classification. After removal of undesired artifacts, segmentation process extracts region of interest from the image. Segmentation breakup in whole CAD process is illustrated in Fig. 2.

In this paper, we present our novel segmentation approach for extraction of lesion from skin in presence of artifact like hairs, water bubbles and vessels by employing luminance enhancement and contrast stretching techniques along with wavelet transform for effective segmentation.

## Related work

**Fig. 3:**
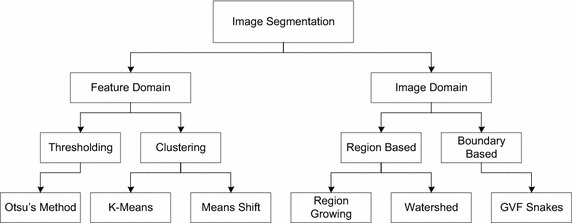
Segmentation classification

In literature, various researches have been conducted to explore the effective segmentation algorithm, therefore variety of of approaches have been already proposed. Mainly, segmentation algorithms could be divided into two groups i.e. feature domain and image domain based methods, which can be further classified as clustering, thresholding, region based and edge/boundary based methods respectively, as shown in Fig. 3. A comprehensive image segmentation survey of skin lesion is provided in Celebi et al. (2010). Soille (2013) proposed an approach based on global and adaptive thresholding along with clustering technique. The proposed approach gives good performance for segmentation of lesion from skin. Though performance may be degraded on the images having poor contract between lesion and background skin. Also, global thresholding produce poor segmentation results due to its basic assumption that image has bimodal histogram. A fully automated segmentation method is based on threshold for dermoscopic images is described in Kruk et al. (2015). This method utilizes histogram based thresholding on all three RGB colors. Celebi et al. (2010) presented a fusion based method. In their proposed method, they applied more than one threshold for a group of images using fusion of four algorithms which includes Huang’s algorithm (1995), Kapur’s algorithm (1985), Kittler’s algorithm (1986), and Otsu’s algorithm (1975). Also, Humayun et al. (2011) proposed a multi threshold algorithm which divide the image histogram iteratively into multiple classes by selecting the threshold for each class using Otsu’s method. In another approach, segmentation is performed by using double threshold (Abbas et al. 2011).

Clustering algorithm groups set of pixels in such a ways that pixels in a group are more similar to each other than the pixel in other groups. Lee and Chen (2014) presented an approach based on fuzzy c-mean clustering (FCM) by using type-2 fuzzy set algorithm (Zadeh 1975). They also utilized the 3D color constancy algorithm to minimize the affects of skin tone variations and shadows in images at pre-processing stage. In another approach, fuzzy c-mean clustering and density based clustering (DBSCAN) is utilized for the segmentation of lesion from background skin on mobile platforms (Mendi et al. 2014).

Simple region-based segmentation method groups or split the adjacent pixels or sub-groups into larger or smaller groups on the basis of some homogeneity criteria. The criteria may be color, texture or average gray levels (Gonzalez and Woods 2002). Region base algorithms are not easy to implement as skin lesion have different variable artifacts like different skin type, water bubbles, skin color variation and hairs, which leads to over segmentation (Gonzalez and Woods 2002). Many region based algorithms have been proposed, which includes multi-scale region growing (Hoffmann et al. 2003), morphological flooding (Soille 2013) and statistical region merging (Lissner and Urban 2012). A detailed comparison of different techniques for segmentation of lesions for dermoscopic images is presented in Gómez et al. (2008). This comparison includes techniques based on thresholding and region based methods. However, they excluded edge-based techniques in their comparative analysis.

Edge-based lesion segmentation methods are generally based on the detection of continuous boundary around the lesion using dynamic contour models or with edge detection algorithms. The edge detection algorithms detects image gradients variation to segment lesion from skin (Gonzalez and Woods 2002; Celebi et al. 2008). Abbas et al. (2011) has presented a technique for lesion boarder detection. In their approach, they utilized least-squares method for edge point detection and dynamic programming(DP) to located the boundary of lesion. Another edge based approach using zero-crossings of Laplacian-of-Gaussian (LOG) is presented in Gonzalez and Woods (2002). There are also a large variety of proposed contour based methods (Day and Barbour 2000; Celebi et al. 2008). A contour based technique based on gradient vector flow (GVF) is presented in Day and Barbour (2000). This method is an extension of active contour or normal snake methods, in which curve is deform by the given energy function. Also, Celebi et al. (2008) presented an approach which uses geodesic edge tracing mechanism to locate the active contour of lesion. Most recently, Abuzaghleh et al. (2015) performs lesion segmentation using active contour algorithm along with parse-field level-set method (Whitaker 1998) for active contour evolution. The performance of edge based methods suffers from the presence of fake edges due to artifacts like hairs, vessels and gel in dermosocpic images. Also in some cases boundary between lesion and background skin is not well not define because of smooth transition between skin and lesion (Schmid 1999).

## Methods

**Fig. 4:**
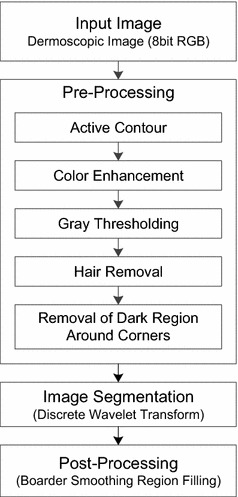
Proposed methodology

In this section, we present the methodology adopted in our proposed approach for the segmentation of lesions from skin in the presence of artifacts like skin lines, vessels, gel and hairs. The proposed algorithm consist of three stages which includes: pre-processing stage for image enhancement along with hair detection/inpainting for artifact removing; segmentation of the lesion area using wavelet based approach and then finally post processing stage for improving segmentation results. The flow diagram of the proposed methodology is presented in Fig. 4. The proposed system takes a dermoscopic image as an input and color enhancement along with thresholding is performed at pre-processing stage. Also hairs, the most unwanted artifact, are removed to improve the segmentation results. This is accomplish by hair enhancement, then segmentation to detect hairs and further removing them by inpainting the hair pixels. The system then performs the detection of four corners to eliminate the undesired details and extract the area of interest for segmentation. In the final step lesion is segmented from the background skin image by using wavelet transformation.

### Input image to system

**Fig. 5:**
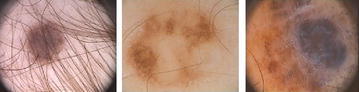
Example images from PH data-set

In this work images from PH2 data-set are used as input to the proposed system. Images are well diversified in nature and contain number of artifacts which can cause the segmentation more challenging. PH data-set contains total 200 dermoscopic images, obtained from Hospital Pedro Hispano database which includes different type of image variations like melanocytic nevi and melanomas. These are 8 bit RGB color images with dimensions 768 × 560 pixels. All experimentation, analysis and results are based on these dermoscopic images, some examples are shown in Fig. 5.

### Image pre-processing

The first step in image segmentation is to prepare the image for segmentation. This is mostly done by applying some pre-processing techniques on the dermoscopic images. The proposed pre-processing stage involves several steps which are described below.

#### Active contour

In this section, we present our technique to define active contour i.e. the active area of interest to work with. This step is required as dermoscopic image contain a rounded background on each corner of image. Active contour is a model which describes the boundaries of shape in an image. It is particularly designed for the problems where the approximate shape of the boundary is already known. However, it also has the few drawbacks such as they are sensitive to local minima state, minute feature are often ignored and their accuracy depend on the convergence policy (Qian et al. 2013). An active contour or a simple elastic snake can be represented by the energy function defined by *n* points $V_{i}$ where $i=0,1,2,3,\ldots ,n$ like

1 $$ E_{snake} = \int _{0}^{1}(E_{internal}(v(s))+ E_{image}(V(s))+ E_{con}(v(s)) ) ds $$

Energy function of snake is sum of its external and internal energy. As internal energy $E_{internal}$ is composed of continuity in contour and smoothness of contour and $E_{snake}$ is the sum of all the forces due to the image itself $E_{image}$ and the constant force introduce by user i.e. $E_{con}$. The basic purpose of contour is to find out the area of interest in order to reduce any unwanted area. In proposed research, area inside the circular boundary is area of interest and thus dark corners around the image can be eliminated by finding the active contour. Figure 8 shows the dark corners around the images that affect the segmentation process. Once the active contour is selected then image is further converted into binary image which is used as a binary mask to extract the area of interest.

#### Color enhancement

In this section, we present our approach for the selection of color enhancement technique which improves segmentation results for dermoscopic images. The most simple method for color enhancement is to find out *Luminance* by linearly combining RGB values into a single value using following formula.

2 $$ Luminance=R\times 0.2989+G\times 0.5870+B\times 0.1140 $$

**Fig. 6:**
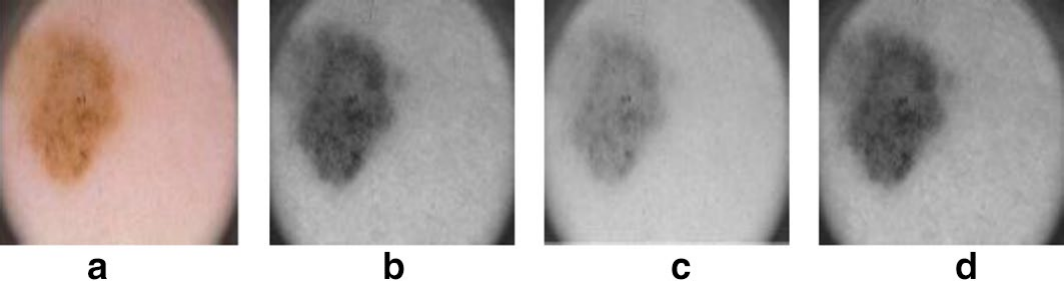
Results of color enhancement techniques: **a** original image, **b** blue color, **c** illumination transformation, **d** highest entropy

Output of different color enhancement techniques are shown in Fig. 6, however after experiments it has been found that blue channel from RGB value produces better results as compare to other techniques described below.

#### RGB with highest entropy

Entropy provide the measurement of image smoothness such that higher the entropy higher the number of gray levels. In this work the entropy of each color is computed on the basis of following formula as:

3 $$ E(c)= \sum _{i=0}^{L-1}p(i)\cdot log_{2}p(i) $$

where *p*(*i*) is the probability of occurrence for each intensity *i* in the image and *c* denotes the respective color channel. After the entropy computation, the RGB color component with highest entropy is selected as:

4 $$ i = arg\,max(E(i)) $$

where $arg\,max(\cdot )$ function returns the argument of maximum entropy and *i* denotes RGB color component.

#### L*a*b color selection

L*a*b color space by breaking it into L, a and b component has also been experimented during analysis. As L*a*b is representation of CIE 1976 (L*, u*, v*) color space where L represent Lightness and a and b are color-opponent dimensions.^1^

#### Blue color selection

This phase analyzes the RBG color space of dermoscopic images for the segmentation of lesion from skin. After the details analysis, color enhancement technique based on blue components is selected for further processing, which gives better segmentation results as compare to other techniques. Therefore blue component is used only because of clear color segmentation between the lesion and normal skin.

#### Gray thresholding

In this approach, image is first converted into gray scale image and then thresholding is applied on the inputted image. In this work Otsu’s method is utilized to find the threshold which exhaustively searches for the threshold that minimizes the intra-class variance as:

5 $$ \sigma _{x}^{2}(t) = \omega _{1}(t)\sigma _{1}^{2}(t)+\omega _{2}(t)\sigma _{2}^{2}(t) $$

where weights $\omega _{1}$, $\omega _{2}$ are the probabilities of the two classes of pixels in an image, which is separated by a threshold t and $\sigma _{1}^{2}$, $\sigma _{2}^{2}$ are variances of these two classes. The purpose of this step is to equally distribute the color along the image and try to accommodate any shadow and higher variation in images which can affect the segmentation process. It is observed during experimentation that thresholding improves the accuracy more than 3–4 %.

### Hair removal

Skin hairs frequently appears in dermoscopic images on background skin. Also, hairs partly covered the lesions which causes interference in reliable lesion segmentation. Therefore, hairs should be detected and excluded from dermoscopic image before the inception of skin lesion segmentation procedure. The hair removal process involves three steps i.e. hair enhancement, hair segmentation and hair in-painting. There are number of hair removing methods discussed in literature (Abbasi et al. 2004). During the experiments it has been found that simple morphological operations and directional filter are simple techniques to be used for the hair removal. Moreover, for morphological processing there is a tradeoff between the image edge blurring and the size of structuring elements (Gonzalez and Woods 2002). Due to this morphological trade-off, hair detecting based on direction filter gives better results.

#### Hair enhancement

Hairs are enhanced before segmentation and removal. To accomplish this task, line directional filters are applied by utilizing the following Gaussian filters as:

6 $$ g(x,y)=  G_{1}(x,y)-G_{2}(x,y)$$

7 $$\begin{aligned} g(x,y) &= k_{1e}^{\left[ -\left( \frac{x^{2}}{2\sigma _{x1}^{2}}+\frac{y^{2}}{2\sigma _{y1}^{2}}\right) \right] }-K_{2e}^{-\left[ -\left( \frac{x2}{2\sigma _{x2}^{2}}+\frac{y2}{2\sigma _{y2}^{2}}\right) \right] } \end{aligned}$$

where $k_{1}$ and $K_{2}$ are constant. The rotation of *g*(*x*, *y*) along angle is given by

8 $$g_{\phi i}(x{'},y{'}) = g(x,y)$$

where $$x{'} = xcos\theta +ysin\theta $$ and $$y{'} = ycos\theta -xsin\theta $$.

**Fig. 7:**
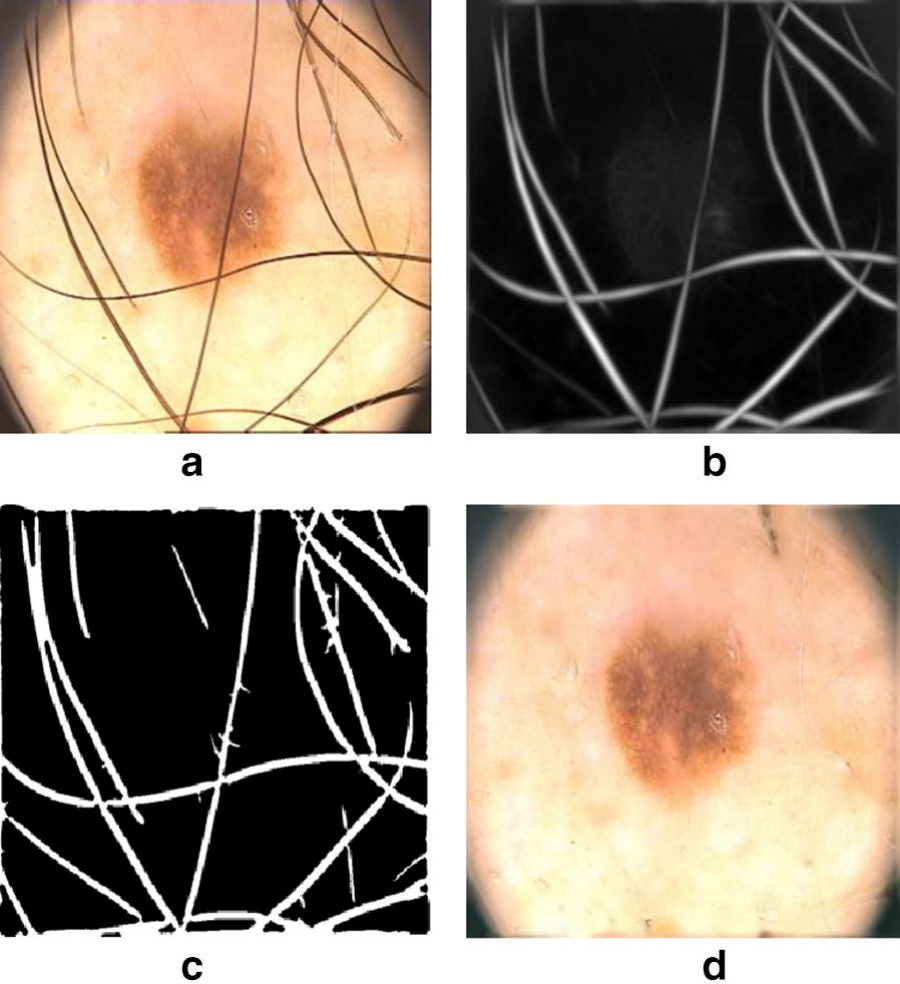
**a** Original image, **b** hair enhancement, **c** hair segmentation, **d** hair inpainting

 The response of each filter for any input image is given by

9 $$R_{i}(x,y) = g_{\phi _{i}}(x,y)\otimes I(x,y)$$

where $\otimes$ is the special convolution. This step is depicted in Fig. 7b.

#### Hair segmentation

To exclude hairs from lesion and background skin, hair segmentation is performed by thresholding the dermoscopic image such that

10 $$ H(x,y) = \Big \{_{1,\quad if\,I(x,y)\,\le\,T_{hair}}^{0,\quad if\,I(x,y)\,>\,T_{hair}} $$

where *H*(*x*, *y*) is binary hair mask.

#### Hair in-painting

After hair segmentation, in-painting is performed to remove hairs and fill pixels with appropriate color information as shown in Fig. 7d. This phase utilizes binary hair mask for in-painting by using PDE based algorithm, which fills the hairs with the values along the level lines called isophotes. We also employ image smoothing to remove any dark spot and slightly remaining unwanted hair pixels. To remove aforementioned noise, we utilized median filter due to its nature of removing noise without blurring the image. It will replace a gray level pixel with median of neighborhood pixels.

11 $$y(i,j) = median\{x(m,n),(m,n)\epsilon w(i,j)\}$$

### Detection of four dark corner

**Fig. 8:**
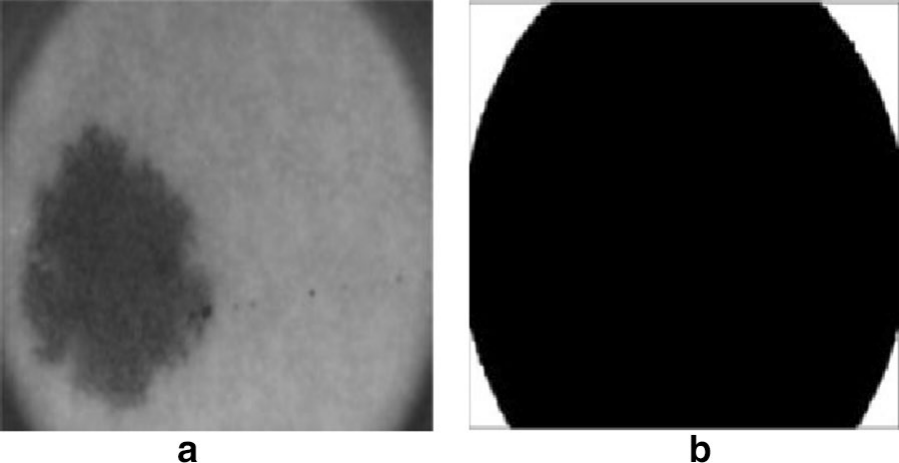
**a** Image with dark corners, **b** binary mask

Most of the dermoscopic images contain black corners which is mainly due to the use round circular lens designed for a smaller sensor in dermatoscope. Therefore image circle can not illuminate a large enough area and thus cause dark corners. Normally these rounded shape dark corners has nearly the same intensity of skin lesion. Therefore, these dark corners must be removed to improve the performance of segmentation algorithm. In order to remove dark corners from the image, we employ thresholding based on Otsu’s method which will make a binary mask for these dark corners. The Otsu’s method divide the image into two classes $C_{1}$ and $C_{2}$ by threshold *k* such that

12 $$C_{0} = \{0,1,2,3,\ldots ,k\}\quad and\quad C_{1}=\{k+1,k+2,\ldots ,L-1\}$$

where L is total number of gray levels in image. The binary mask created by this method is used to remove the dark corners from dermoscopic image. Figure 8 shows the dark round corner dermoscopic image along with a binary mask generated using Otsu’s method based thresholding.

### Segmentation using wavelet transform

After hair detection and in-painting, processed dermoscopic images still may contains some artifacts like water bubble and gel. In this work, we employ discrete wavelet transformation (DWT) for segmentation of lesion from skin in presence of aforementioned artifacts. Wavelet transforms utilizes the similar approach like Fourier transform. It converts the signal into frequency domain and also provides the time resolution for the converted signal. Instead of decomposition of signals into sum of sin and cosine functions, wavelets decomposes into wavelet coefficients. Variety of DWT flavors exits in literature known as mother wavelet families. The DWT is like a sub band system in which signal is decomposed into details H (Horizontal), V (Vertical) and D (Diagonal) and A (Approximation) bands.

**Fig. 9:**
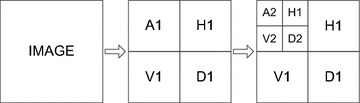
Wavelet transformation

Approximation is the image approximation remaining after removing the details. Then details of image is further divided into the horizontal details, vertical details and diagonal details. Approximation can further divided to to next level of approximation and details as shown in Fig. 9. Where H1 gives the horizontal detail, D1 gives the diagonal detail, V1 gives the vertical detail and A1 gives the approximation detail which could be further decomposed to next level. This property of wavelet could be used for image segmentation in medical imaging.

**Fig. 10:**
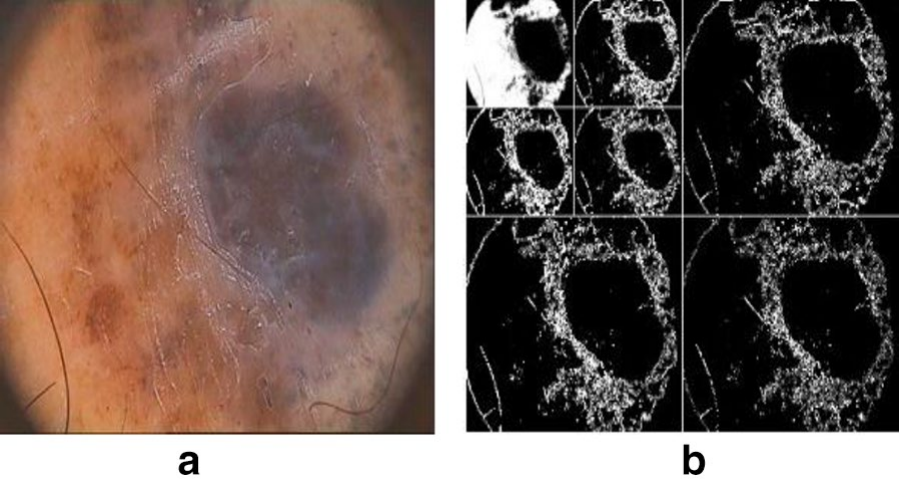
Wavelet transformation on dermoscopic image: **a** original image, **b** wavelet transformation

Moreover, wavelets are being used in image processing for de-noising, edge detection, segmentation, compression, encoding and decoding. In this work, we are interested in image segmentation whereas most of the early work in wavelets are on texture based analysis therefore many texture based segmentation methods exists in literature. However, in case of dermoscopic images most of the proposed works exploits wavelet transform fuzzy algorithms (Castillejos et al. 2012). Recently, Sadri et al. proposed a new approach for the segmentation of skin lesion by using wavelet networks (Sadri et al. 2013). Here, wavelet transform is utilized for the segmentation as well as denoising of input image. It has been observed during the experiments that wavelet are very useful in case of removing some artifacts on dermoscopic images like water bubbles and gel effects. Even hairs are broken into minute fragments which can be remove by applying morphological operations as illustrated in Fig. 10.

In this work, detail qualitative analysis is performed for the selection of suitable mother wavlet family. The Cohen–Daubechies–Feauveau biorthogonal wavelet is selected and applied on the blue channel of pre-processed image because its demonstrate superiority as compared to other mother wavelet families. During experimentation second level approximate wavelet component gives best results on the inputted image. Although, different combinations are tried by combining two components with different orientation but it has been found that the best results are being obtain through approximation.

### Post processing

After the wavelet transformation, post processing operations are performed to find the final segmented binary result, by keeping large connected binary objects and joining adjacent binary regions. As the processed image at this stage may contain holes due to the intensity difference in skin lesion image. Therefore, morphological operations are performed to fill holes and remove any extra elements other than the skin. The regions belongs to the dark corners around the image is removed by the binary mask in pre-processing step. However the small isolated islands are kept and joined together if they are very near to skin lesion. On the other hand, islands far away from skins lesion are removed by morphological erosion and dilation operations.

**Fig. 11:**
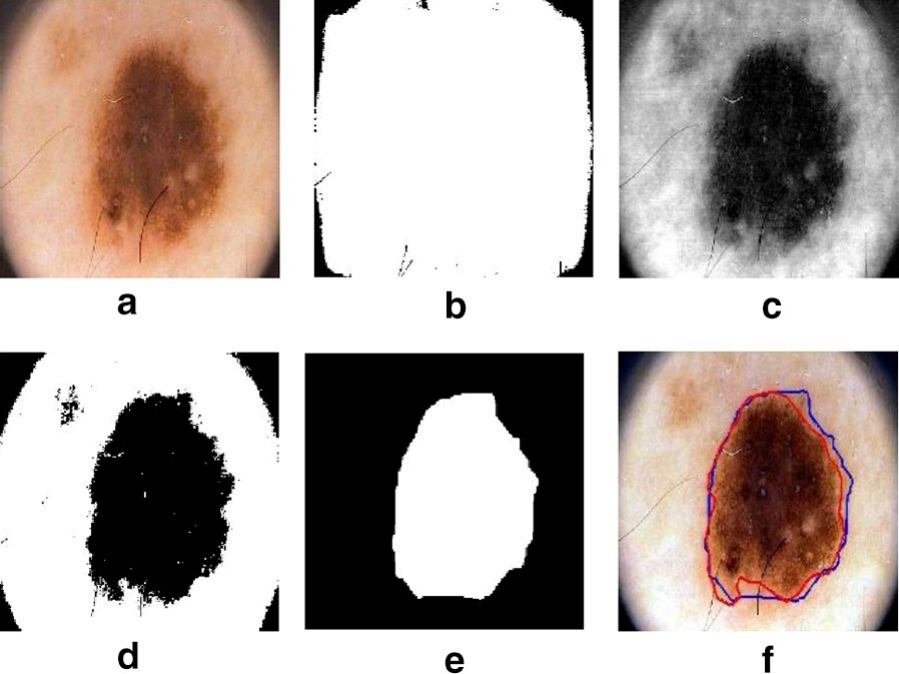
Steps of the proposed dermoscopic image segmentation algorithm **a** original image, **b** active contour, **c** blue channel, **d** wavelet transformation, **e** segmented image after post-processing, **f** dermoscopic image with segmentation

Finally, the segmented binary lesion images have ragged boundaries which requires smoothing. This can be done by the convolution filter but in this work smoothing operation is performed by using average filter.

13 $$ I_{out}(i)=\frac{1}{W}\sum _{j=-(w-1)/2}^{(w-1)/2}I_{int}(i-j) $$

where $I_{out}$ is input boundary coordinated and *W* is the filtering degree. Figure 11 illustrates the overall process for segmentation of dermoscopic images.

## Result and discussion

This section contains the detail of different experiments, their setup and results. Various experiments are performed to find out the effective approach for the segmentation of lesion from skin in the presence of undesired artifacts like gel, hairs and water bubbles. Here we present the dermoscopic images dataset and their inclusion criteria including base line metrics. Afterwords experiments performed and relevant procedure adopted during those experiments will be discussed.

The initial requirement of experiment was identification of reliable source for dermatology images. Thus, dermoscopic images for analysis are acquired from Hospital Pedro Hispano database which are 8 bit RGB images of size 768 × 560 pixels. The ground truth is also available with manual segmentation for the comparison with automated segmentation. For the selection between different options in experiment 1, we utilized 45 images out of 200 images. However, segmentation of dermoscopic images by using proposed technique is done in experiment 2 and experiment 3 is performed to compare the acquired result with already existing approaches.

### Evaluation metric

In proposed work, three baseline matrices are used for the qualitative analysis and comparison. These three metrics are: average true detection rate (ATDR), average false positive rate (AFPR), and average error probability rate (AEP).

ATDR is average rate of true detection rate (TDR) which is the rate of pixels identified as skin lesion by both the manual and automated segmentation this can be explained mathematically as:

14 $$ TDR = \frac{\sharp (SR\cap GT)}{\sharp GT} $$

where *SR* is segmented results by automated system and *GT* is ground truth by manual segmentation. And average is a mean on all the TDR of each image.

AFPR is average rate of false positive rate (FPR) which is the rate of pixels identified as skin lesion by automated system and which is marked as no-lesion by manual segmentation results. FPR can be explained mathematically as:

15 $$FPR = \frac{\sharp (SR\cap \overline{GT})}{\sharp GT}$$

AEP is average error probability (EP) which is define as:

16 $$EP = \frac{FPR+FNR}{TPR+FPR+TNR+FNR}$$

where false negative rate (FNR) is the rate of pixels not identified as skin lesion by automated system and which is marked as lesion by manual segmentation results. FP can be expressed mathematically as

17 $$FNR = \frac{\sharp (\overline{AS}\cap GT)}{\sharp GT}$$

and true negative rate (TNR) is define as the rate of pixels which are not identified as the lesion type by both automated system and manual system.

18 $$TNR = \frac{\sharp (SR\cap GT)}{\sharp GT} $$

and true positive rate (TPR) is define as the rate of pixels which are truly identified by both the automated and manual system.

19 $$ TPR = \frac{\sharp (\overline{SR}\cap \overline{GT})}{\sharp \overline{GT}}$$

Furthermore, we have also used dice similarity coefficient (DICE) to show the accuracy of proposed segmentation results. This similarity measure is calculated between automated generated skin lesion binary mask (ABM) and the manual binary mask (MBM) which are given alongwith the database. Equation 20 shows the mathematical expression for DICE.

20 $$ DICE = \frac{2~Area (ABM \cap MBM)}{Area (ABM)~+~ Area(MBM)} $$

### Experiment 1: Qualitative analysis for the selection of image enhancement approach

The first step in image segmentation is to convert the color image into gray scale. In our case we have experimented with different options to convert the 8 bit RGB image to gray scale image. Different option includes the simple gray scale conversion, L*a*b color, RGB color and gray level thresholding to produce an image with fine details.

**Table 1:** Qualitative analysis for gray level selection

RGB color selection	ATDR	AFPR	AEP
Simple gray conversion	73.0731	3.013	8.7125
R value from RGB scale	52.793	0.5045	13.576
G value from RGB scale	78.772	3.3987	7.8718
B value from RGB scale	94.74	3.8341	5.6915
L value from L*a*b scale	69.7847	2.8542	9.47
a value from L*a*b scale	21.9443	53.4237	62.0178
b value from L*a*b scale	36.4974	53.7768	60.7508

First image is tested with simple gray level conversion with different thresholds, then image RGB color space is analyzed by applying the same threshold value. After that image is converted into the L*a*b color space and each color component is tested separately. The analysis conducted in experiments shows that B values gives the better results as compared to other techniques as well as RGB scales as depicted in Table 1.

### Experiment 2: Qualitative analysis for the selection of wavelet family

The purpose of this experiment is to analyzed the performance of different wavelet families for the segmentation of skin lesion. In this work, the following mother wavelets are implemented:

- ‘haar’ or ‘db1’ Haar
- ‘db4’ 4th order Daubechies
- ‘sym4’ 4th order Symlets
- ‘bior6.8’ Cohen–Daubechies–Feauveau biorthogonal
- ‘jpeg9.7’ Antonini–Barlaud–Mathieu–Daubechies

Details could be found at^2^:

In this experiment, we employed different wavelet families for the segmentation of dermoscopic images from PH dataset. Moreover, the performance is evaluated by computing the statistical measure like ATDR, AFPR and AEP for each wavelet family. And it is obvious from the Table 2 that Cohen–Daubechies–Feauveau biorthogonal wavelet attains the higher value of ATDR along with lower values of AFPR and AEP as compare to other wavelet families. Therefore, Cohen–Daubechies–Feauveau biorthogonal wavelet proves his superiority for the segmentation of lesion from background skin.

**Table 2:** Qualitative analysis for wavelet selection

Wavelet selection	ATDR	AFPR	AEP	DICE
Haar	93.195	5.8823	5.8006	92.13
Db4	93.182	5.8616	5.8106	92.09
bior6.8	93.87	5.4718	5.2751	92.72
jpeg9.7	93.4152	5.8751	5.7813	92.57

**Fig. 12:**
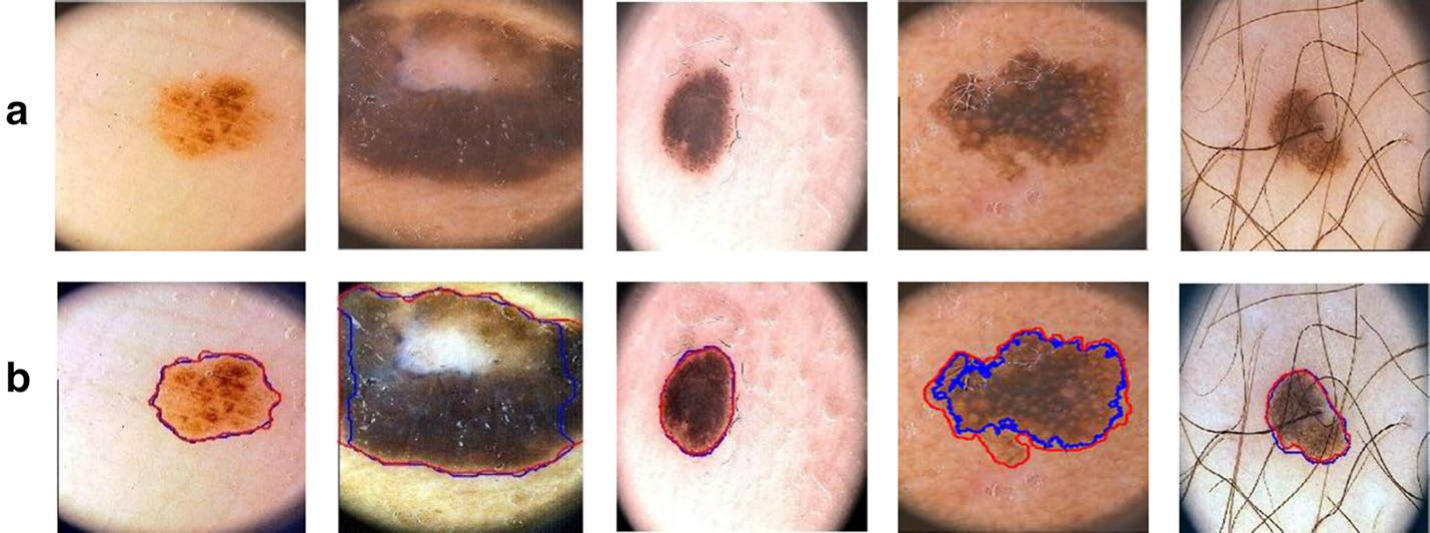
**a** Original images, **b** images with segmentation

### Experiment 3: Qualitative analysis for segmentation of skin lesion

In this experiment, we compare the proposed approach for the segmentation of dermoscopic images on PH dataset with existing approaches proposed by Silveira et al. (2009). The statistical measures like ATDR and AFPR are computed and compared with the results of segmentation methods proposed in Silveira et al. (2009), as illustrated in Table 3. The eminence of proposed approach is indicated by the obtained results. This is mainly due to the induction of color enhancement and hair removal steps in proposed method.

**Table 3:** Comparison of proposed method with existing approaches

Technique	ATDR	AFPR
Discreate wavelet transformation	93.87	5.43
Adaptive thresholding (AT)	91.30	4.08
Gradient vector flow (GVF)	90.89	8.67
Level set method of Chan et al. (C-LS)	83.39	2.55
Fuzzy-based split-and-merge (FBSM)	93.67	3.73

Some of the segmentation results of proposed approach on dermoscopic images in presence artifacts like water bubble, hairs and gel effect are shown in Fig. 12. The red outline around the lesion represents the manual segmentation performed by dermatologist while blue outline shows the segmentation achieved by the proposed approach. The segmentation results from the figure clearly demonstrate the effectiveness of proposed approach in the presence of artifacts like veins, hairs, water bubble and gel effects. However, contrast between skin and lesion with similar level of color and intensity variations may cause under and over segmentation which is insignificant. Also artifacts like water bubbles and gel effect in dermoscopic images are removed automatically during segmentation stage with the use of wavelet transformation.

## Conclusion

In this paper, we have proposed a DWT based approach for the segmentation of nevus and melanocytic lesions from background skin using dermoscopic images. The analysis is carried to select the appropriate image enhancement technique for segmentation problem. In this regard, techniques like color Entropy, luminance transformation and channels like L*a*b color space with RGB color space are analyzed. However, blue channel is found to appropriate for further processing. Also the proposed technique caters the problem of most unwanted artifacts like hairs and small vessels which are enhanced by using line directional filters and then PDE based in-painting algorithm is employed on detected pixels representing these artifacts. The four dark corners in dermoscopic images are also removed to because it improves segmentation results significantly. In addition to this, segmentation is carried out using Cohen–Daubechies–Feauveau Biorthogonal Wavelets because it produces better results as compared to others like Haar, db4 or sym4. The most significant outcome of using wavelets is the removal of certain artifacts with less effort, e.g. hairs, bubbles and skin tones. The segmented lesion image is enhanced using morphological operations in post processing stage. The proposed methodology is tested on dermoscopic images of PH dataset and achieves ATDR of 93.87 % and AFPR 5.43 %. The experimental results of proposed approach shows the effectiveness of wavelet based segmentation for lesion from background skin.

## Future work

In order to achieve the accuracy in CAD systems for skin lesion larger dataset is needed. Secondly classification of skin lesions is also not mentioned. Ground truth of images also need to be performed by multiple doctors to reduce its subjectivity and the final ground truth should be established based on these multiple varying judgments.

In case of segmentation, hybrid techniques can be introduced by combining wavelet based segmentation approach with other techniques. Secondly, some new concepts are also emerging such as curvelets which can be explored for segmentation problems. Moreover, better technique of hair detection and removal can enhance the segmentation results.

## Footnotes

1 http://www.umsiko.co.za/links/color.html.

2 http://wavelets.pybytes.com/wavelet/sym4/.
